# Rapid Testing System for Rice Quality Control through Comprehensive Feature and Kernel-Type Detection

**DOI:** 10.3390/foods11182723

**Published:** 2022-09-06

**Authors:** Huma Zia, Hafiza Sundus Fatima, Muhammad Khurram, Imtiaz Ul Hassan, Mohammed Ghazal

**Affiliations:** 1 College of Engineering, Abu Dhabi University, Abu Dhabi 59911, United Arab Emirates; 2 National Center of Artificial Intelligence-Smart City Lab, NED University of Engineering and Technology, Karachi 75270, Pakistan

**Keywords:** food quality assessment, rice quality control, machine learning, computer vision, rapid testing

## Abstract

The assessment of food quality is of significant importance as it allows control over important features, such as ensuring adherence to food standards, longer shelf life, and consistency and quality of taste. Rice is the predominant dietary source of half the world’s population, and Pakistan contributes around 80% of the rice trade worldwide and is among the top three of the largest exporters. Hitherto, the rice industry has depended on antiquated methods of rice quality assessment through manual inspection, which is time consuming and prone to errors. In this study, an efficient desktop-application-based rice quality evaluation system, ‘National Grain Tech’, based on computer vision and machine learning, is presented. The analysis is based on seven main features, including grain length, width, weight, yellowness, broken, chalky, and/or damaged kernels for six different types of rice: IRRI-6, PK386, 1121 white and Selah, Super kernel basmati brown, and white rice. The system was tested in rice factories for 3 months and demonstrated 99% accuracy in determining the size, weight, color, and chalkiness of rice kernels. An accuracy of 98.8% was achieved for the classification of damaged and undamaged kernels, 98% for determining broken kernels, and 100% for paddy kernels. The results are significant because the developed system improves the local rice quality testing capacity through a faster, more accurate, and less expensive mechanism in comparison to previous research studies, which only evaluated four features of the singular rice type, rather than the seven features achieved in this study for six rice types.

## 1. Introduction

Rice is a staple food for more than half of the world’s population [1]. As a primary crop, it contributes a dominant proportion of the balanced diet of humans, predominantly in the diet of Asians, where most of the world’s rice is consumed and grown [2]. Pakistan is the 3rd largest exporter (80%) and the 12th largest producer of rice worldwide. Rice constitutes the second largest cash crop, and about 10% of its agricultural land is dominated by rice crops [3]. Therefore, high-quality rice production is a significant source of food, income, and economic growth [2]. Considering the continuing advancement of knowledge and technology in quality control, coupled with consumers’ food quality expectations, the need for precision and transparency in quality monitoring has become more important. Therefore, evaluating rice based on quality attributes is an extremely pivotal step to achieve and maintain high quality for both consumption and to maximize economic return [4].

There are typically seven physical parameters associated with rice quality: damaged, broken, paddy, colored (yellow and white), chalky, stonesand foreign objects, and dimensions/weight of the rice kernel [5]. The quality of rice kernels is often compromised by the quality of the seed and working parts of the post-harvest agricultural processing machinery.

Generally, in parts of the world where rice is cultivated, and more specifically in Pakistan, the investigation of the type of grain, grading, and gauging quality attributes to national and international standards is done manually by human inspectors (vernier, caliper, and weighing scales) [6]. The labor-intensive checking process is multifaceted. It depends on human factors, for example, the number of people in the crew and the efficiency of performing a particular task. Although it ensures accuracy to some degree, it requires significant trained manpower, resulting in high labor costs, and judgments are still subjective. Therefore, the quality of the kernels is manually checked every 1–2 h as a new batch is introduced into the warehouse [7].

In agriculture, it is noteworthy that traditional visual and manual equipment-based (Vernier, caliper, and weighing scales) quality inspection systems, if replaced by computer vision systems at the commercial level, could prove to be fast, efficient, and accurate evaluation systems [4]. Computer vision-based determination of the qualitative characteristics of rice kernels will allow industrialists to adopt a non-destructive and automated continuous food assessment inspection system [1]. This will allow processing operations to be monitored continuously, thereby enabling an operator to react quickly to changes in kernel material properties, thus reducing the overall manual inspection cost [8] while improving accuracy. 

In this study, techniques using image processing and computer vision, combined with machine learning, were used to assess grain quality using a non-destructive and inexpensive approach. This study presents Pakistan’s first commercial automated rice quality assessment system. The uniqueness of our system compared with other computer vision-based rice quality assessment systems is that it detects all seven major physical features of the rice kernel that play a significant role in assessing and controlling the quality of the dry kernel. In addition, the system was deployed in different rice factories for a period of approximately 3 months to evaluate its efficacy. The testing indicated a successful implementation in the operation of rice factories, associated with the quality of the rice kernels. It was found that 99% accuracy was achieved for the size, weight, color, and chalkiness. For the other parameters, a precision of 98.8% was achieved for the classification of damaged and undamaged kernels, 98% accuracy for spotting broken kernels, and 100% for paddy rice kernels. Based on these findings, it was deduced that the system is efficient in analyzing the quality of kernels in IRRI-6, PK386, 1121 white rice, selah rice, super kernel basmati brown rice, and white rice based on their specific characteristics. The rice types selected for this study were based on their importance, as they are the most significant varieties produced, consumed, and exported internationally by Pakistan. 

A detailed review of previous works related to computer vision and machine learning technology involved in food grain classification is presented to grade the novelty of our system with the systems developed in the past. 

### 1.1. Deep Neural Networks

Deep learning neural networks are popular and effective for dealing with image classification problems in machine learning-based algorithms. They do not rely on a feature extraction algorithm because they use image pixels as input and can produce high accuracies [8]. Ref. [4] addressed the performance of four different algorithm frameworks to classify processed rice into their corresponding classes of low-processed sound kernels (LPS), low-processed broken kernels (LPB), high-processed sound kernels (HPS), and high-processed broken kernels (HPB). The artificial neural network (ANN) algorithm, which has a 12 − 5 × 4 topology, was very effective with an accuracy of 98.72%. Ref. [2] used convolutional neural networks (CNN) for the classification of two different types of rice, that is, whole rice and broken rice, based on their different sizes. The data consisted of two thousand camera images of broken and whole rice of the Loc troi strain. When the CNN model was applied, it exhibited a precision of 99.16% and 89.75% for the training and testing data, respectively. The study was conducted on only one rice type, making it inefficient to assess the quality attributes of other types of rice that are mostly produced and consumed in the region.

### 1.2. Support-Vector Machines (SVM) 

Support-vector machines (SVM) are supervised learning models that are classified based on arranging the input vectors into a high-dimensional space and building a hyperplane for segregating the data. It is an effective technique for solving classification and pattern recognition problems [9]. Ref. [10] employed a multi-class SVM to classify and grade various varieties of rice in their respective classes with an accuracy of 86%. The SVM comprising the universal Pearson VII kernel function successfully classified processed rice into corresponding classes with an improved precision of 98.48%. Previously, [11] an attempt was made to develop an SVM algorithm based on a linear kernel to effectively separate overlapping rice kernels. Contour detection and watershed algorithms were used to evaluate the contours and perform segmentation, respectively. The calculated classification accuracy was 88.0%, whereas that of the segmentation was 96.0%.

### 1.3. Fuzzy Inference System

Machine learning techniques based on fuzzy logic have been proven to successfully interpret human behavior in terms of judgment and analysis [12]. An intelligent method based on fuzzy logic was developed to qualitatively grade milled rice by utilizing the AND operator to generate 25 rules in the rule base of the fuzzy inference system. Compared with skilled workers, the overall confidence was 89.80%, and high sensitivity and specificity were observed for grading rice into its respective classes. Ref. [13] provides more recent evidence that confirms the capabilities of fuzzy logic-based systems as they implemented another automatic rice classification system (ANFIS). They claimed that the system outperformed the k-NN (k-nearest neighbor algorithm) and SVM ML methods with an accuracy of more than 98.5% in classifying broken and whole-grain rice.

### 1.4. k-Nearest Neighbor Algorithm (k-NN)

Owing to its simplicity, effectiveness, and nonparametric nature, the k-nearest neighbor algorithm (k-NN) is also popular for classification problems. It groups similar points together and determines classification boundaries based on the proximity of neighboring points [14]. Experiments for grading Paw San rice into three classes, A, B, and C, were conducted [15] prior to segmentation, image pre-processing was performed on images captured against a dark background, and selected features were fed to k-NN classifiers. The resulting accuracies were as high as 100% for class A, 93% for class B and 83% for classes A, B, and C, respectively.

### 1.5. Edge Detection, Segmentation, and Thresholding Algorithms

Recently, Ref. [16] proposed an evaluation system to inspect the quality of Type C4 Raja rice using a digitally processed Canny edge detection algorithm. The length-to-width ratio was calculated to determine the rice category. Ref. [17] are among the few researchers that have addressed the issue of rice grain evaluation by implementing a typical webcam for image acquisition. The bounding box and band detection techniques were executed to identify objects by creating an enclosing boundary and computing their area for discerning specific regions. One of the criteria for analysis was the overlapping and non-overlapping positioning of grains, where the enumerated error for overlapping was quite high at 53.82%, whereas that of non-overlapping was significantly low at 0.47%.

The objective of our study is to qualitatively analyze rice kernels based on computer vision and machine learning techniques. The study successfully classified seven different rice types, IRRI-6, PK386, 1121 white rice and selah rice, basmati brown super kernel, and white rice, to the best of our knowledge. Each rice type was classified by detecting all the physical features (damaged, broken, paddy, colored (yellow and white), chalky, stones, objects, and weight) that are responsible for grading the rice product on the basis of quality. The images were acquired from a flatbed scanner to extract the morphological characteristics of the rice, such as size (length, width), color, and weight, to assess the chalkiness, yellowness, damage, and whether they were paddy or not. Random forest, linear regression (LR), and visual geometry (VGG-16) were the primary models used in this study. LR works on the basis of probability to form an algorithm based on predictive analysis and is commonly used for classification applications. It uses a sigmoid function to map the predicted values in the form of probability [18]. We believe that there is significant potential to develop methods based on LR and VGG16 models. As reported for [19] both LR and VGG-16, rice varieties in Thailand have been classified.

They claimed to have chosen VGG-16 because it showed good results in a short span of time. However, to obtain better results, they had to significantly reduce the image size, compromising the efficiency of the model, whereas the LR model also yielded only satisfactory results. Ref. [8], in their work for rice classification, used the “inrange” function, which defined upper and lower boundaries to classify rice type on the basis of color, but the accuracy achieved was lower than that reported in our study using a similar technique. We further developed a user-friendly and non-complex graphical user interface (GUI) system that is missing in many studies [9,19,20], making them impractical.

The application provides the system with a provision to be customized, which would allow the end-user to input data and custom parameters, as every manufacturer has different standards for broken and paddy rice, chalkiness, and yellowness.

A detailed review of previous works is presented in Section I. The proposed system design methodology is described in Section II. The vetting and results are discussed in Section III. Section IV presents the conclusions of this study.

## 2. Proposed Methodology

**Figure 1:**
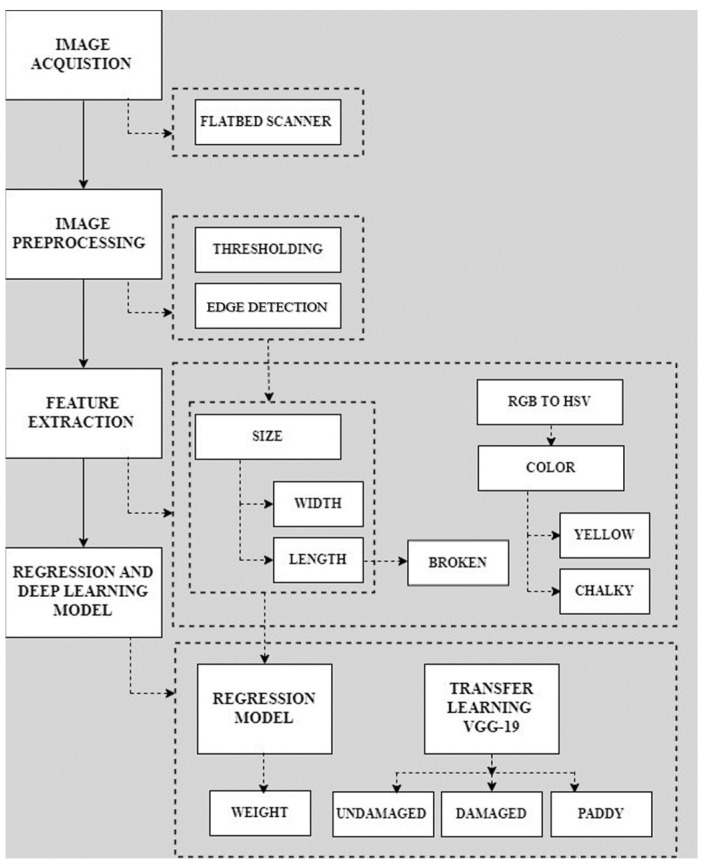
The systems flow diagram.

This study successfully presents an image-processing-based automated classifier system for classifying rice on the basis of quality. The hardware and software parts consist of a flatbed scanner and machine learning models, respectively, the working processes of which are described in detail in the following sections. The flow diagram of the system is shown in Figure 1.

## 3. Preparation of Dataset

### 3.1. Features of the Flatbed Scanner 

To begin the study, the dataset was collected from rice industrialists, and the quality assessment was performed using manual tools, Vernier calipers, which are commonly used to measure the length and width of the rice kernel. Each industrialist suggested its own thresholds for color and broken length. A Canon Cano Scan LIDE 220 scanner was used to capture two-dimensional images of rice kernels with its contact image sensor (CIS). The scanner offers high-resolution pictures up to 4800 × 4800 dpi, enabling it to capture crisp text and clear photos with remarkable accuracy, and with its 48-bit internal color depth, detailed colors are observed. It accommodates cloud compatibility, requires nominal cabling, and offers a touchscreen.

### 3.2. Calibration of Flatbed Scanner

**Figure 2:**
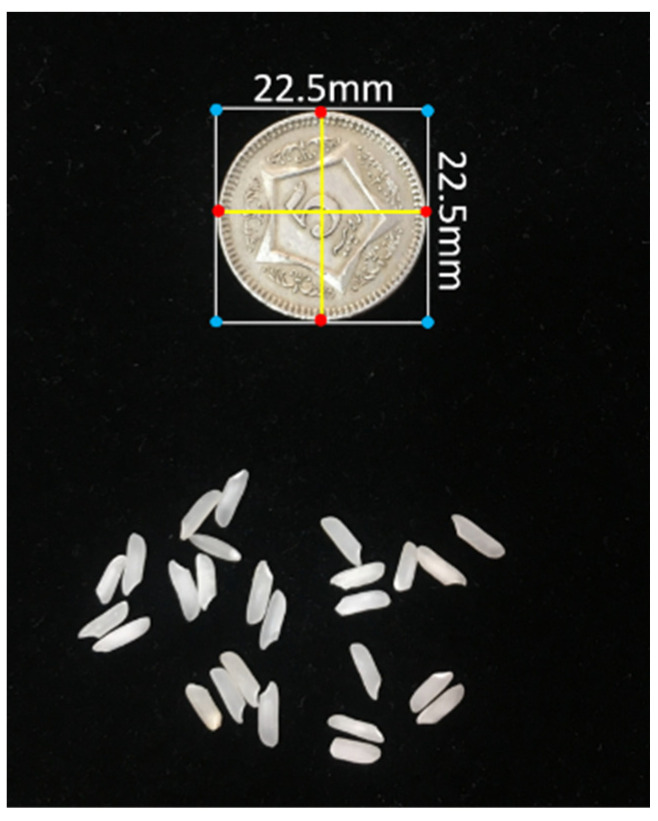
Calibration of the scanner using a coin.

The scanner was calibrated using the pixel per metric (ppm) method, where an object of known dimensions was taken as a reference object and kept at a known position; in this case, the left-most corner was selected. A coin of 22.5 mm diameter was placed on the scanner, and Equation 1 was used to convert it into a number of pixels. The calculated ppm was 12.50. Figure 2 shows the scanner calibration.

(1) $${\mathrm{pixels}}_{{\mathrm{per}}_{\mathrm{metric}}}=\frac{\mathrm{Reference}\;\mathrm{object}\;\mathrm{width}\;\left(\mathrm{pixels}\right)}{\mathrm{Known}\;\mathrm{width}\;\left(\mathrm{mm}\right)}\;\;.$$

## 4. Image Analysis and Processing

### 4.1. Image Sample Preparation

Initially, the model was trained on images of IRRI-6 rice kernels due to the fact that it contributes to about 80% of rice production and is the most exported rice in Pakistan. They are long, non-basmati rice with a soft texture, mainly produced in the region of Sindh and the southern geographical areas of Pakistan. Prominent characteristics included a typical 6.4 mm length, 14% moisture content, and 3% chalkiness.

**Figure 3:**
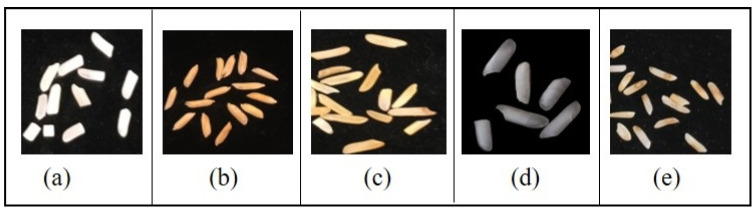
Visuals of (**a**) broken, (**b**) paddy, (**c**) yellow, (**d**) chalky, (**e**) damaged rice kernels.

To predict the weight of rice kernels, a dataset of 2500 kernels was prepared and manually weighed using a weighing scale. For the prediction of damaged and paddy rice, the model was trained and tested on a dataset of 12,000 rice kernels, divided into two portions, one for spotting damage and one for paddy kernels. A total of 80% of the acquired dataset was selected for training the model, and 20% for testing. Figure 3 shows the visual features of rice kernels.

### 4.2. Image Acquisition 

**Figure 4:**
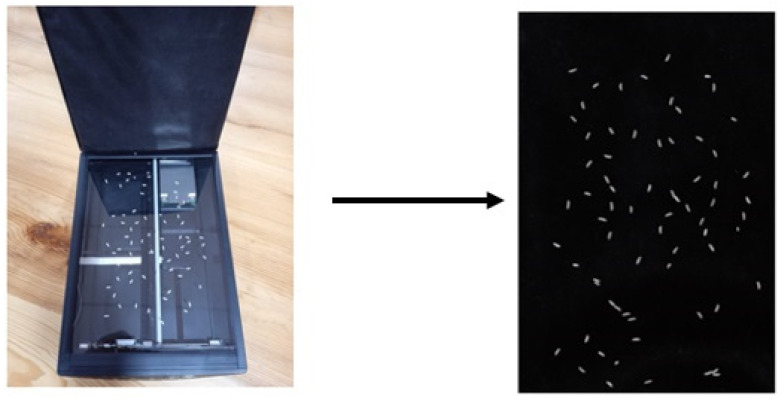
Image of the rice sample acquired from a calibrated flatbed scanner.

The quality testing process of the rice kernels was initiated by placing the rice sample on the flatbed scanner. The kernels were distributed in a single layer on the glass plate of the scanner and covered with a black sheet, such as paper or cloth, in order to capture an image in the reflected mode. Figure 4 shows an image of the rice sample acquired using a calibrated flatbed scanner.

### 4.3. Image Pre-processing and Smoothing

**Figure 5:**
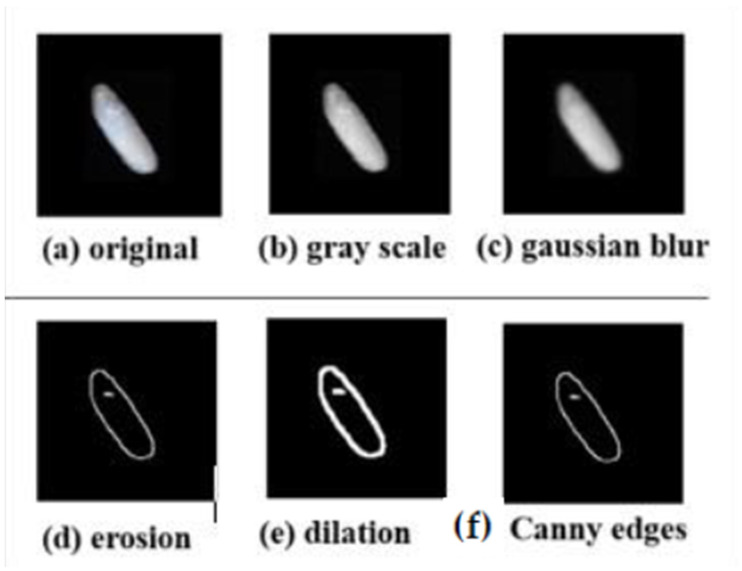
Image pre-processing stages for feature extraction of rice kernel.

**Figure 6:**
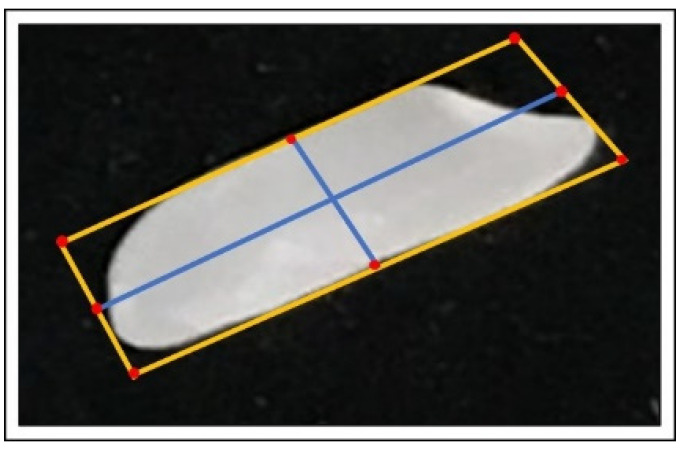
Each rice kernel is labelled with their boundaries. The boundaries highlighted in orange after the contours were detected successfully.

Prior to the implementation of the model, Python’s OpenCV library, which is typically used in computer vision applications to process images, was employed. The acquired images were converted to grayscale to reduce the amount of data, as it enables complex procedures to be accomplished in less time, and noise removal is performed via the application of a Gaussian filter, which blurs the images by removing detail and noise to avoid the detection of these points as edges later. Several edge detection methods, such as Canny, differentiation, thresholding, and linking, have been used to discern rice kernels. Therefore, gray scaling, binary thresholding Canny edge, and contour detection methods were employed on the images during pre-processing. The image pre-processing stages applied to the rice kernel are shown in Figure 5. Figure 6 shows the boundaries highlighted in orange after the contours were detected successfully.

## 5. System Design 

### 5.1. Feature Extraction

The rice sample is a combination of whole, damaged, broken, paddy, colored (yellow) chalky, stones, and objects. These seven parameters, including the weight of the rice kernel, are typically associated with rice quality attributes. The following sections provide a brief explanation of the methodology used to extract each feature.

### 5.2. Morphological Features of the Rice Kernel (Width and Length)

**Figure 7:**
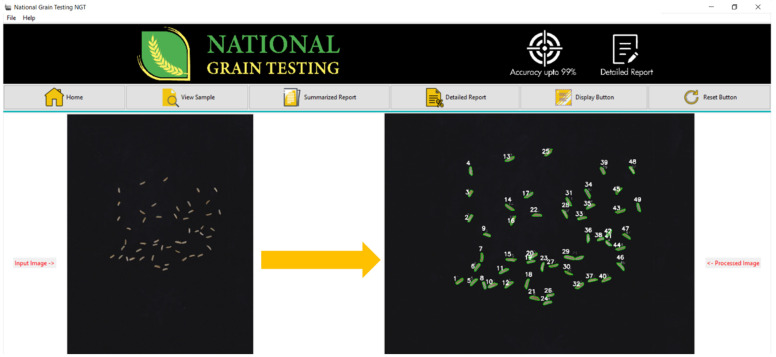
Computes the size (length and width) of rice kernel.

After the contours are detected by image pre-processing, a rotated bounding box is created around that area, and its coordinates are arranged in the top-left, top-right, bottom-left, and bottom-right. The outline of the object is shown in orange, and the vertices are highlighted as red dots. Next, the midpoints between the four vertices were computed. Red dots are drawn at the midpoints on the image followed by connecting them with blue lines, as shown in Figure 6. By connecting these midpoints and calculating the Euclidean distance, the corresponding length and width of the rice kernel is calculated. Figure 7 shows highlighted boundaries of the rice kernels.

### 5.3. Rice Weight

Weight is predicted by random forest using length and width as the input variables. A dataset of two thousand and five hundred rice kernels was prepared and weighed manually using a weighing scale. The rice industry measures the broken kernel percentage by weight rather than the number of rice kernels. This is the most decisive feature in rice quality analysis, as rejection is based on the amount of broken rice as a percentage by weight to the standard rice percentage by weight.

Random forest is a supervised machine learning technique with a decision tree (DT) as the basic building block [21]. A decision tree is a logical model in which the training data are split based on their similarities. Every node in a DT consists of a logical expression that is used to decide whether a data point is sent to an adjacent node located on the left or right side of the given node [22]. The first node of the decision tree is called the root and the succeeding splits are called branches. The end nodes where data cannot be split any further are called leaves. To make a prediction with a trained DT, the input data are supplied at the root node, from where the data flow through the branches based on logical expressions until they reach a leaf node. The output of a DT is an average of linear regression of the training data points corresponding to the leaf node, and the [13] shape and parameters of a DT are highly dependent on the training dataset. The performance of any regression model is typically quantified using the root mean square error (RMSE), mean absolute percentage error (MAPE), and correlation coefficient (R^2^), as shown in Equations (2) and (3). The actual value and the predicted quantity are represented by ‘A’ and ‘B’ for ‘n’ number of predictions.

(2) $$\mathrm{RMSE}=\sqrt{\frac{1}{n}\;\overset{n}{\underset{i=1}{\sum }}\;{\left(\mathrm{Ai}-\mathrm{Bi}\right)}^{2}}$$

(3) $$\mathrm{MAPE}=\frac{1}{n}\sum_{i=1}^{n}\frac{\left|\mathrm{Ai}-\mathrm{Bi}\right|}{\mathrm{Ai}}\times 100.$$

To predict the weight of the rice kernels, the dataset was split into two features and the decision was made according to the performance of the tree based on the RMSE and MAPE of the testing dataset.

### 5.4. Yellow and Chalky Rice Kernels

Yellowness is an undesirable trait of milled and paddy rice kernels that typically occurs under humid conditions or when drying is delayed. The grains turn yellow or discolored and worsen over time. However, chalkiness, characterized by an opaque area on the rice grain, is an unwanted quality that discourages consumers from purchasing it and is also believed to have a lower starch content compared to normal rice kernels.

**Figure 8:**
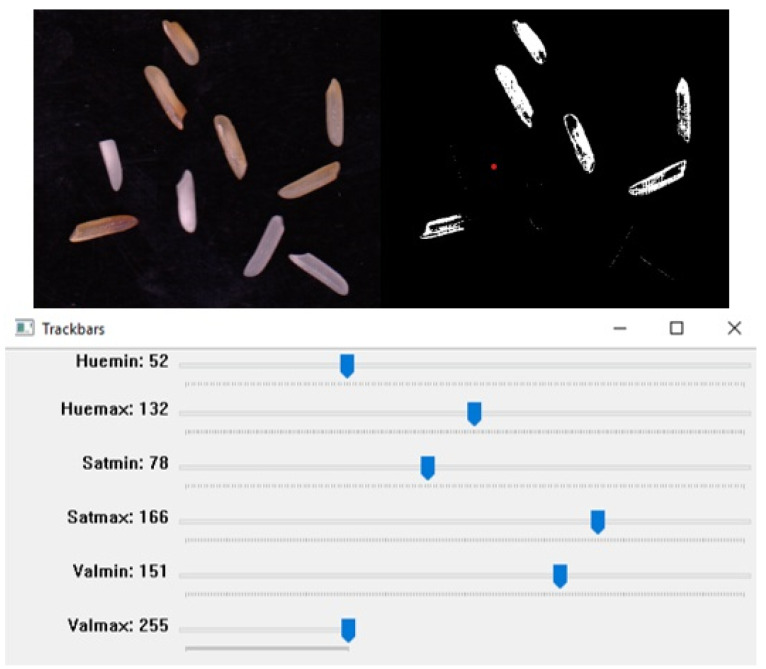
The image shows rice images before and after scanning and the ranges of given HSV components extracting yellow rice from the sample.

The Python inRange function was used to calculate the degree of yellowness and chalkiness of the rice kernels. First, we converted our images to the HSV format, as images could be captured in inconsistent lighting. HSV comprises three components: hue, saturation, and value, and it represents the human perception of colors better than RGB. A track bar was used to set the HSV values for yellow and white colors, as they could vary depending on the type of rice used. A mask was created using the InRange function and subsequently applied to the image. In the example shown in Figure 8, we analyzed the yellowness in the rice grains by adjusting the ranges of hue, saturation, and value accordingly. For chalkiness, the same work was performed for color classification.

### 5.5. Damaged Rice and Paddy Rice

**Figure 9:**
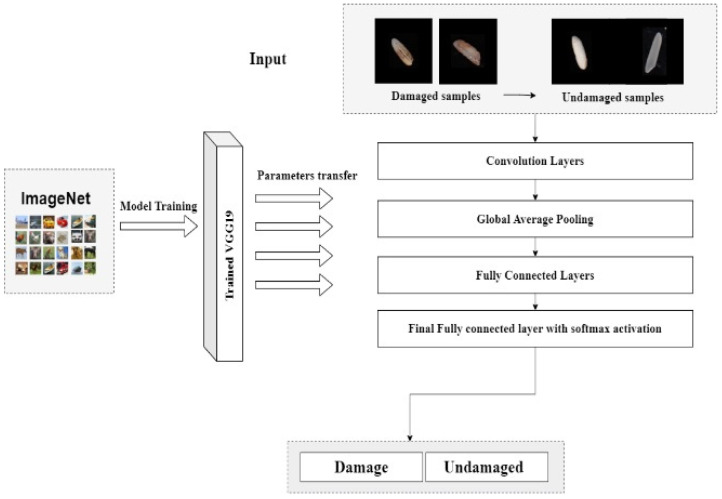
Process diagram of the transfer learning model VGG19.

Damaged rice kernels are fully or partially darkened as a result of insect, mold, water, or heat damage. We prepared our own dataset to detect damaged rice kernels. The data were divided by rice industrialists into two portions of 3000 rice kernels. A transfer learning method for processing and a pre-trained model named VGG19 were employed. Convolutional neural network (CNN) layers were also added, and the dataset was trained in binary mode. Figure 9 shows the process diagram of the VGG19 model. Paddy rice kernels distinguished by the hull surrounding the rice grain were also detected using an analogous approach to prepare the dataset and model implications, as used to distinguish damaged and undamaged rice kernels from the sample.

Transfer learning is a deep learning approach that utilizes relative pre-trained models to develop new solutions, unlike other application-specific machine learning methods [8]. It is mainly applied in natural language processing and computer vision applications, which require large datasets for training models. As the weights of the old domain or task are transferred to the new task of interest, transfer learning is highly beneficial in applications with limited or insufficient data [20]. The pre-learned patterns aid in making a new task efficient by transferring the maximum amount of pre-gained knowledge, which is also the ultimate goal of transfer learning. This method works by retraining some layers of the new model, while keeping the other layers the same. This helps retain and reemploy the labelled data [4]. One of the main requirements of this method is to provide the same input size for both the tasks. In the case of different inputs, a pre-processing step is added to equalize the new input size with that of the old one. The number of layers to be repurposed and retrained varied with each application. We chose the deep learning model VGG-19 (Visual Geometry Group-19) on the basis that it is used for large-scale image recognition and possesses an excellent model architecture; it is also known as the Oxford Net [8]. The model is a 19-layer deep convolutional neural network (CNN) and is considered an excellent vision model. The default input size for the VGG19 model was 224 × 224 pixels, with three channels for RGB images. It has convolution layers of a 3 × 3 filter with a stride of 1 and a max pool layer of a 2 × 2 filter of stride 2.

### 5.6. Broken Rice

**Figure 10:**
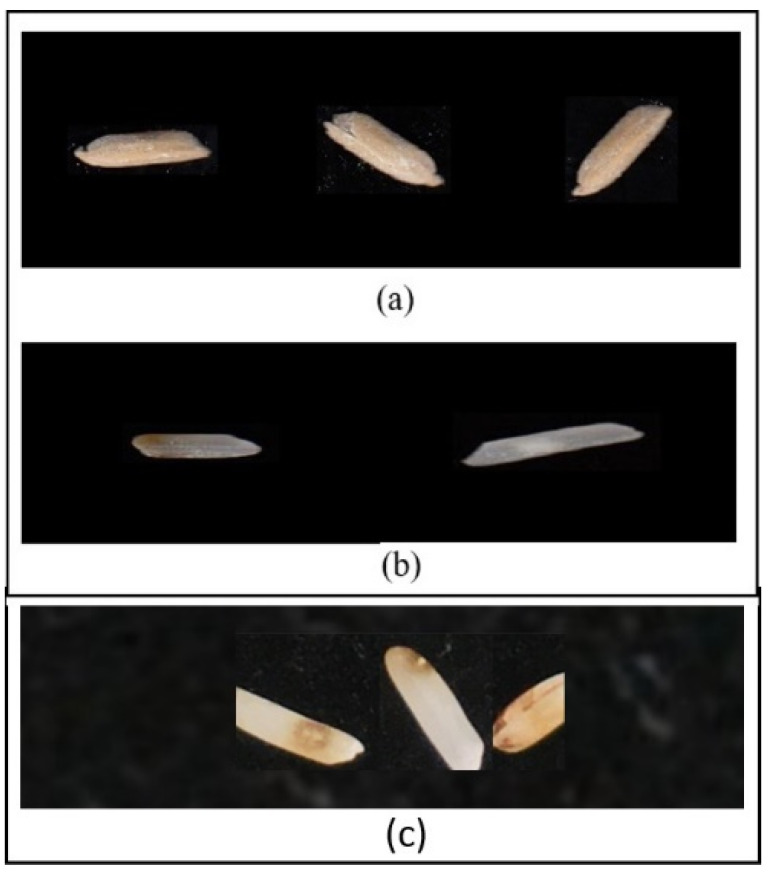
(**a**) shows spotted paddy rice and (**b**) shows spotted damaged on the left and undamaged on the right side of the image, and (**c**) shows broken rice kernels.

Broken rice can result from the drying, transport, and milling processes. A threshold of broken rice length is set; any grain shorter than that value is classified as broken, whereas above that value, the grains are considered unbroken. The threshold depends on each rice type; therefore, the seller can adjust the threshold based on their requirements. Figure 10 shows the spotted paddy, damaged, and broken rice kernels.

### 5.7. Additional Measurements

The average grain length (AGL), average grain width (AGW), and other details, such as head rice AGL and AGL, were also calculated using the system along with whole grain count, broken grain count, total weight, and total number of rice kernels. Head rice is a rice kernel with a length greater than that of the broken rice kernel. Additionally, a detailed analysis of rice length (in mm), including information such as how much rice has a length in the range of 1 mm to 2 mm, 3 mm to 4 mm, is extracted and provided. It also provides its AGL and weighted percentages, as shown in Table 1. Table 2 provides a manual and software comparison of the average grain length.

## 6. Results and Discussions

**Figure 11:**
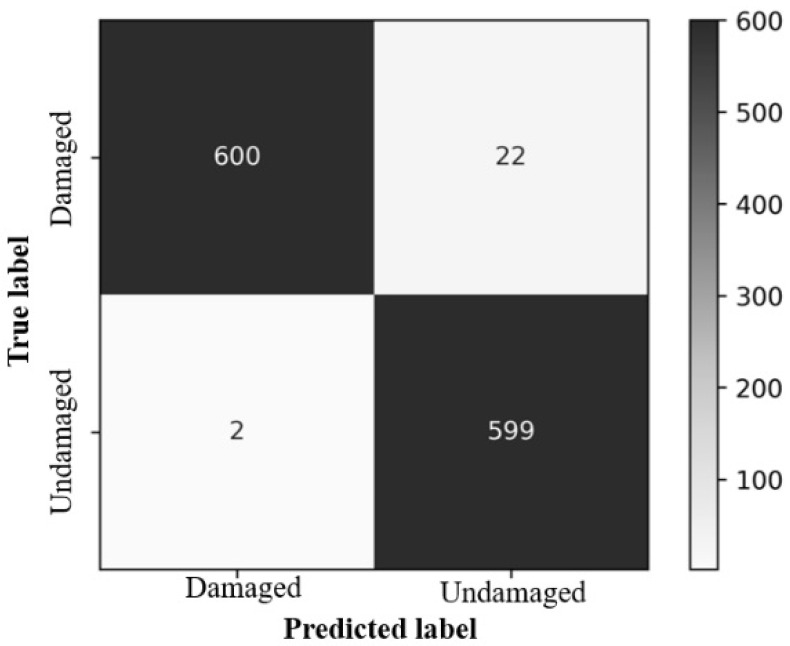
Confusion matrix of the damaged rice kernels.

**Figure 12:**
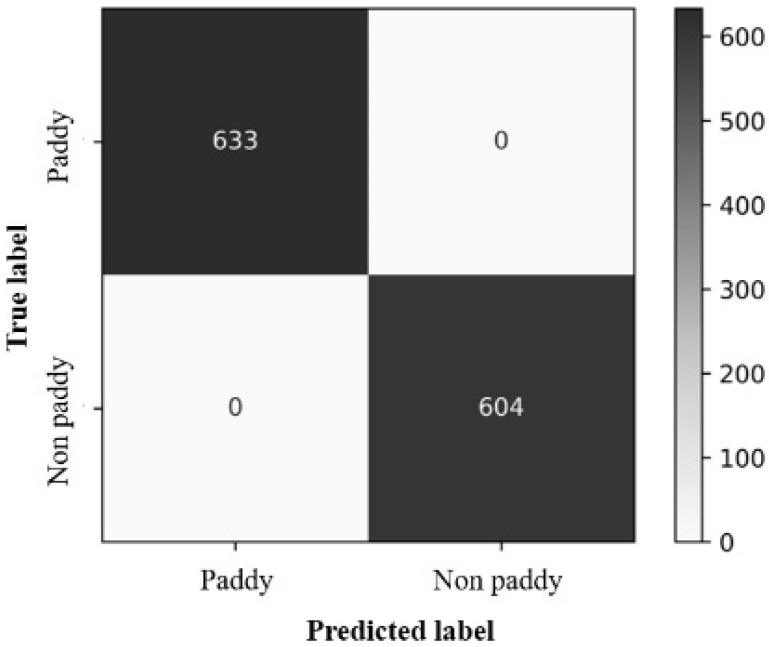
Confusion matrix of the paddy rice kernels.

The results of the system were validated by cross-checking with the conventional method of measuring the characteristics associated with the quality of the rice product. A digital Vernier caliper was used to calculate the length and width of various grains to determine the average difference between the values calculated by the system and those calculated manually. For length, the difference is 3–4 percent, that is, up to 0.324 mm, while for width, it is approximately 10 percent, which is 0.208 mm. Yellowness is determined using the inRange function, where threshold pixels are set to distinguish yellow from rice grains, yielding 99% accuracy. However, when the process was performed to detect chalky rice after readjusting the HSV color space, we obtained an accuracy of 99%. The VGG-19 machine learning technique was used to evaluate damaged and paddy rice kernels, and the model spotted object features with efficacies of 98.8% and 100%, respectively. Figure 11 and Figure 12 show the system generated confusion matrices of the damaged and paddy rice kernels, respectively.

**Figure 13:**
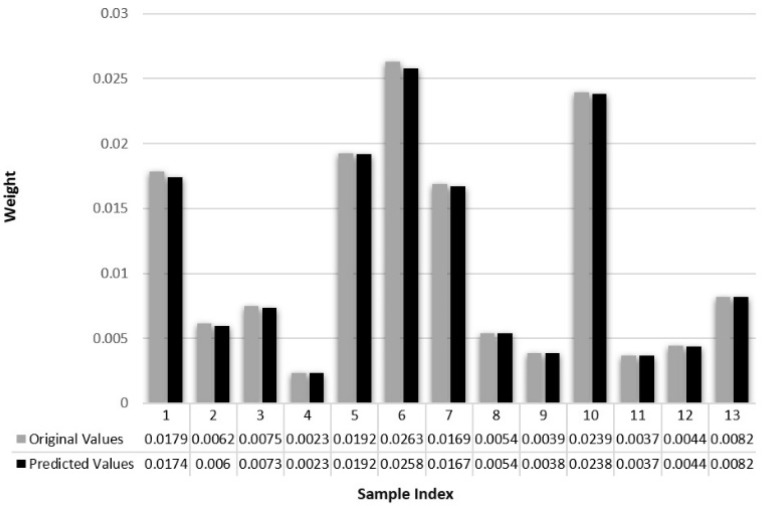
Graphical representation of the predicted and true weight values of the rice kernel.

To predict the weight of the kernel, a random forest regression model was employed; the dataset was split into two features, no trees (estimators) were 100, and the decision was made according to the performance of the tree based on the RMSE and MAPE of the testing dataset, which was 1.21 × 10^−8^ and 6.7 × 10^−5^, respectively. Based on the values of RMSE and MAPE, the system showed 99% accuracy. Figure 13 shows a graphical representation of the predicted and true weight values of the rice kernel.

The identification of broken rice from the dataset was carried out by setting threshold values for the major axis length of the rice kernel, and the generated results of the system were equated with the broken values obtained from the rice industries that showed 98% accuracy.

**Figure 14:**
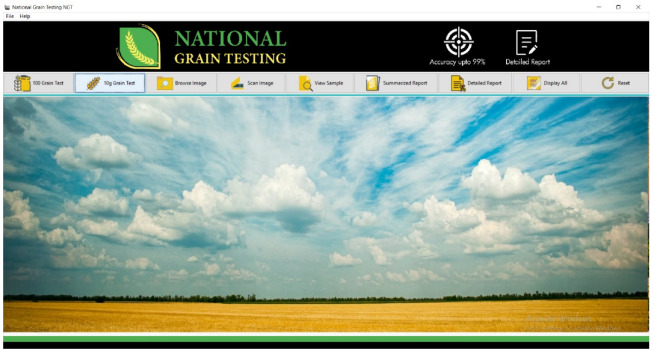
Front-end view of the desktop application.

**Graphical User Interface (GUI):** Python 0.1.9.2 decanter was utilized to make the desktop application, called National Grain Tech; this comprises an interactive design providing a user-friendly environment and features, such as multiple buttons/options to resize the data, and caters for the needs of a new user. Figure 14 shows the front-end view of the desktop application.

**Figure 15:**
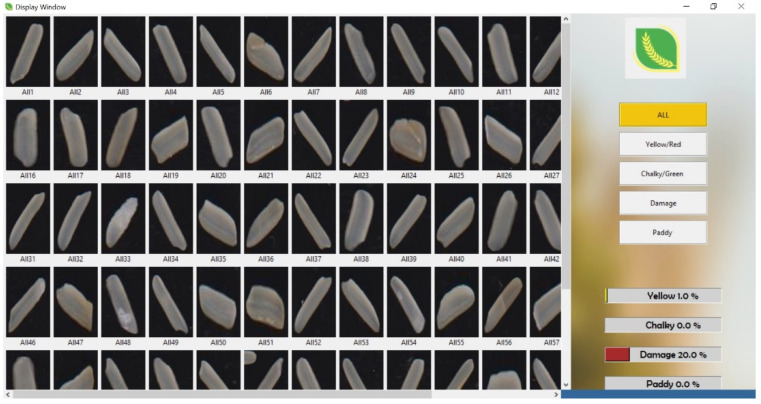
National Grain Tech - generated quality analysis image.

The application is user-friendly and easy to use by non-technicians. The applications have the option to scan and run the test for 10 g or 100 g rice samples. It provides options for a detailed and summarized test report. Figure 15 presents the summarized test results for a sample of each rice kernel and indicates the percentage of features such as yellow, chalky, damaged, and paddy rice. The sample contained 20% damaged rice. When selecting the particular feature tab, the particular rice kernels identified according to that feature are displayed.

**Figure 16:**
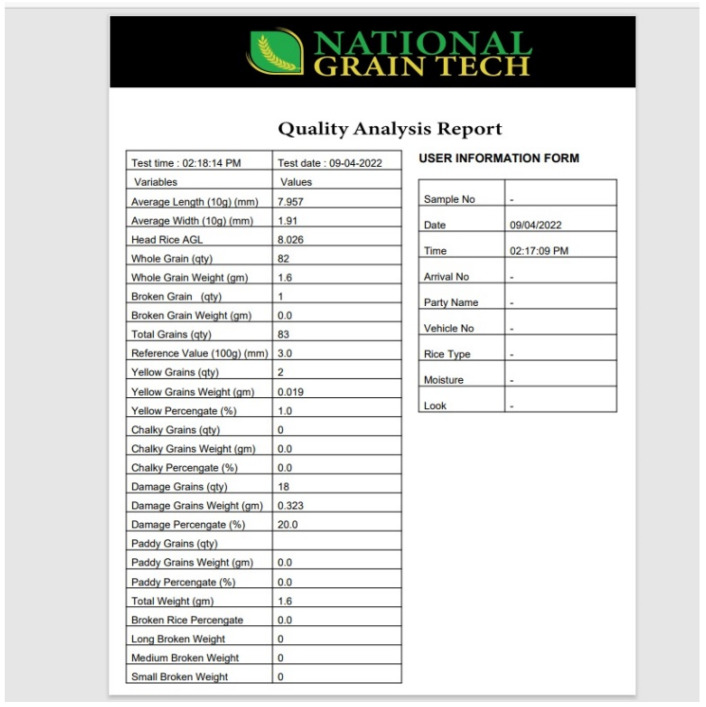
National Grain Tech-generated report.

Figure 16 shows the detailed quality analysis report generated by the application specifying total number of grains, average length and width, weight and number of grains for yellow, damaged, paddy, and chalky rice.

To the best of our knowledge, this is the first time a rice quality analyzer has been able to achieve such high accuracy while providing a rapid and comprehensive feature analysis. To test the developed system, it was deployed in different rice factories for approximately 3 months to evaluate its efficacy. The testing indicated a successful implementation in the operation of rice factories, associated with the quality of the rice kernels. Work is still underway to adapt this to large-scale implementation in Pakistan’s rice industry.

Table 3 summarizes the accuracy achieved for all features. The achieved accuracy for determining features, such as size, weight, color, and chalkiness, was 99%. Damaged and undamaged rice kernels were detected with 99.8% accuracy. In addition, broken and paddy rice kernel features were detected with 98% and 100% accuracy, respectively.

## 7. Conclusions

Overcoming the challenges faced by rice industries due to the existing traditional manual assessment of rice quality, which is prone to errors, is tedious and time-consuming. This study presents Pakistan’s first AI-based rice quality analyzer (‘National Grain Tech’) that analyzes the quality of a sample of rice kernels in less than 60 s. The system was successfully tested on six different rice types (IRRI-6, PK386, 1121 white rice, selah rice, super kernel basmati brown, and white rice) and was able to predict seven major features that strongly contribute to the quality assessment of the rice products, which is a unique result. Previous research has only evaluated singular rice types and extracted a maximum of four features. The results demonstrated 99% accuracy in determining the size, weight, color, and chalkiness of rice kernels, whereas an accuracy of 98.8% was achieved for the classification of damaged and undamaged, 98% accuracy for spotting broken, and 100% for determining paddy rice kernels. The system proved to be non-destructive and precise, enabling an operator to react quickly to changes in comparison to conventional methods that are inconsistent, unreliable, and inefficient. The results are significant as the developed system improves local rice quality testing and quality control capacity through a faster, more comprehensive, accurate, and less expensive mechanism in comparison to previous research studies. This system was validated by deploying and testing it in various rice factories for a period of approximately 3 months to evaluate its efficacy. The testing indicated a successful implementation in the operation of the rice factories, improving the testing time and accuracy, resulting in precise quality assessment, which significantly impacts and determines the price and export potential of the tested rice. Based on the advice of rice experts in the industry, we plan to extend this work to include the detection of insects, moisture detection, and spotting of overlapped kernels by incorporating advanced techniques.

## Figures and Tables

**Table 1:** Average length of the rice kernels from the sample and its weighted percentage.

Range (mm)	Count	Average Length (AGL)	Percent by Weight
(1–2)	0	0	0.0
(2–3)	0	0	0.0
(3–4)	2	3.842	1.0
(4–5)	4	4.444	4.0
(5–6)	8	5.572	10.0
(6–7)	44	6.489	73.0
(7–8)	6	7.239	9.0
(8–9)	0	0	0.0
(9–10)	0	0	0.0

**Table 2:** Comparison between manual- and software-measured AGL results.

No of Grains	Average Length (Manual)	Average Length (Software)	Percent Deviation from Manual to Software
100	5.996	6.146	−2.44%
200	5.63805	5.816	−3.06%
300	5.9025	6.044	−2.34%
400	5.789525	5.951	−2.71%
100	5.63805	5.82	−3.13%
100	5.941	5.816	2.15%
200	5.9025	6.09	−3.08%
300	5.9025	6.065	−2.68%
400	5.789525	5.954	−2.76%

**Table 3:** Prediction accuracy achieved for the seven features of rice.

Feature	Achieved Accuracy
Size, weight, color, chalkiness	99%
Damaged and undamaged	99.80%
Broken	98%
Paddy	100%

## Data Availability

The data presented in this study are available on request from the corresponding author.
